# Sofosbuvir-based regimens in the treatment of patients with chronic hepatitis C virus infection: Real-world efficacy in Thailand

**DOI:** 10.1371/journal.pone.0229517

**Published:** 2020-02-27

**Authors:** Apichet Sirinawasatien, Thanaya Techasirioangkun

**Affiliations:** Division of Gastroenterology, Department of Medicine, Rajavithi Hospital, College of Medicine, Rangsit University, Bangkok, Thailand; National Taiwan University Hospital, TAIWAN

## Abstract

**Aims:**

To analyze the efficacy and safety of sofosbuvir (SOF)-based regimens in Thai patients with chronic hepatitis C virus infection who had pre-existing significant liver fibrosis.

**Patients and methods:**

This was a retrospective cohort study, conducted between 1 June 2018 and 31 May 2019 at Rajavithi Hospital, Bangkok, Thailand. All patients completed 12 weeks of SOF-based regimens and had follow-up for at least 12 weeks after therapy discontinuation. The primary outcome was sustained virological response (SVR) 12 weeks after the end of therapy.

**Result:**

A total of 185 patients were included, with 52, 63 and 70 taking SOF+Ledipasvir (SOF+LDV), SOF+LDV+ribavirin (RBV) and SOF+Pegylated interferon (Peg-IFN)+RBV (SOF+Peg-IFN+RBV) respectively. Genotype (GT) 1 was predominant at 40.0%, followed by GT3 at 37.8%, and GT6 at 22.2%. Overall 95.1% of patients in this study achieved SVR (n = 176/185), and the only factor associated with SVR was HCV genotype (p = 0.001). GT6 patients had lower SVR rates compared to GT1 and GT3 patients (82.9%, 98.6%, and 98.6% respectively) while there was no association between SVR and other factors (p >0.05) such as gender, age, BMI, underlying cirrhosis, baseline HCV viral load, or prior treatment history. No serious adverse events were reported in the present study.

**Conclusion:**

Sofosbuvir-based regimens in the treatment of patients with chronic HCV infection were highly efficacious with excellent safety and tolerability profiles in a real-world setting; however, further research is required to establish whether or not such a regimen is an adequate treatment for all genotype 6 patients.

## Introduction

The hepatitis C virus (HCV) was discovered in the late 1980s[1] and was found to be a major cause of cirrhosis and hepatocellular carcinoma. The worldwide population infected with HCV is estimated at 71 million individuals,[2] while in Thailand its incidence is 1–2% of the population.[3] There are seven genotypes (GT) of HCV, and these are distributed unevenly worldwide. Although GT1 is the most prevalent in the western world,[4] in Thailand, HCV GT3 is most common at 46.1%, followed by GT1, GT6 and GT2 at 32.5%, 20.9%, and 0.5% respectively.[5]

For many years, combination therapy of pegylated interferon (Peg-IFN) and ribavirin (RBV) has been the cornerstone treatment for HCV infection. It has been found to achieve a low rate of sustained virological response (SVR) of about 40% in HCV GT1 infected patients and 60–80% of HCV GT2 or 3 infected patients. [6, 7] Its adverse effects can be substantial, however, including flu-like symptoms, psychiatric disorders, and hematologic effects, making it intolerable for a considerable number of patients.[8]

Recently, the introduction of direct-acting antiviral (DAA) agents have revolutionized the treatment of HCV, demonstrating high efficacy and good tolerability [9–12] Since 2018, two DAA agents, sofosbuvir (SOF) and ledipasvir (LDV), have been available in Thailand’s government health system. The Thai government offers these drugs without cost for prioritized patients at a significant stage of liver disease, in accordance with the national health policy for treating chronic hepatitis C. All-oral DAA regimens, which are the treatment of choice for the vast majority of HCV-infected patients, are expensive and therefore cannot be supplied to every patient in our country because of resource constraints; consequently, a SOF+Peg-IFN+RBV combination therapy for 12 weeks is still the standard of care for patients infected with in HCV-GT3, who are interferon supersensitive,[12–14] regardless of underlying liver cirrhosis. On the other hand, for patients infected by HCV-GT1, GT2, GT4, and GT6, the standard regimens for those without liver cirrhosis is SOF+LDV combination therapy for 12 weeks and for those with liver cirrhosis SOF+LDV+RBV combination therapy for 12 weeks in Thailand’s government reimbursement system.

Sofosbuvir, the NS5B nucleotide polymerase inhibitor, has pan-genotypic activity, thus SOF-based regimens have been widely used for the treatment of HCV-infected patients with high rates of SVR at week 12 after completion of therapy (SVR ≥90%) in clinical trials.[14–17] An SVR is associated with a 99% chance of being HCV RNA undetectable during long-term follow-up and can, therefore, be considered a virologic cure for HCV infection.[18] Unfortunately, data on the efficacy of SOF-based regimens in HCV-infected patients are still limited in Thailand. The aim of this study was to evaluate the efficacy and safety of SOF-based regimens for HCV-infected patients. To the best of our knowledge, this is the first report in a real-world cohort of HCV-infected patients treated with SOF-based regimens in Thailand.

## Methods

### Study design

In this retrospective cohort study, conducted from 1 June 2018 to 31 May 2019 at Rajavithi Hospital, a tertiary referral center in Bangkok, Thailand, we analyzed data consecutively from the medical records of all the chronic hepatitis C patients who were treated with SOF-based regimens in the out-patient clinic. The study was carried out in accordance with the ethical principles of the Declaration of Helsinki and was approved by the ethics committee of Rajavithi Hospital (No. 157/2562).

### Participants

The inclusion criteria were patients who: (i) were aged between 18–70 years; (ii) were diagnosed as having HCV infection by detection of HCV RNA in plasma; (iii) had pre-existing significant liver fibrosis (≥F2) determined by ultrasound-based vibration-controlled transient elastography (VCTE; FibroScan^®^) ≥7.0 kPa[19] or histopathological staging ≥F2 stage by METAVIR scoring system; and (iv) had not received previous HCV treatment (defined as treatment-naïve), or had been non-responsive to a previous treatment with Peg-IFN+RBV combination therapy (defined as treatment-experienced).

We excluded patients who: (i) had hepatitis B virus (HBV) or human immunodeficiency virus (HIV) co-infection; (ii) had history of alcohol abuse or abstinence from alcohol for less than 6 months; (iii) had severely decompensated cirrhosis defined by Child-Turcotte-Pugh (CTP) score >9 or model of end-stage liver disease (MELD) score >18; (iv) had severe renal impairment with glomerular filtration rate (GFR) <30 ml/min; (v) were in a state of pregnancy or lactating; (vi) had active underlying disease e.g. autoimmune disease, major depression, thyroid dysfunction, and other severe comorbid diseases; (vii) were diagnosed with hepatocellular carcinoma or other malignancy at the baseline of treatment; or (viii) missed follow-up visit at the end of treatment and/or 12 weeks post-treatment.

### Severity of liver disease

To evaluate the degree of liver fibrosis using a non-invasive tool, VCTE is the most studied radiologic method and is reliable for staging liver fibrosis in HBV- or HCV-infected patients and other liver diseases,[20–24] with a >90% negative predictive value for ruling out liver cirrhosis.[19,25] All patients in the present study had VCTE performed within not more than 6 months before beginning the antiviral treatment by a single experienced operator (Thanaya Techasirioangkun, RN) using a Fibroscan^®^ device (Echosens, France).

Significant fibrosis (≥F2) was defined as VCTE ≥7.0 kPa (PPV: 83–97%, NPV: 23–85%) and cirrhosis was defined as VCTE ≥13.5 kPa (PPV: 52–85%, NPV: ≥95%), in accordance with the Thailand practice guideline for the management of chronic hepatitis C 2018 and the Australian Liver Association expert consensus recommendations.[19] Thus, we classified the patients into two groups, namely a non-cirrhosis group and a cirrhosis one, based on the different treatment regimens for these patients. Baseline CTP and MELD scores were calculated for patients with cirrhosis.

### Treatment regimens

In this study, all patients completed 12 weeks of SOF-based regimens therapy, and they had follow-up for at least 12 weeks after therapy discontinuation. The patients were considered compliant with therapy if they did not miss more than 20% of all prescribed antiviral medication.

HCV-GT3 infected patients were treated with triple-drug therapy (SOF+Peg-IFN+RBV) for 12 weeks regardless of whether they had underlying cirrhosis. SOF dose of 400 mg (MyHep^®^, Mylan Laboratories Limited) was given orally once daily in the morning, Peg-IFN alfa-2a dose 180 mcg (Pegasys^®^, Roche Pharmaceuticals) or Peg-IFN alfa-2b dose 1.5 mcg/kg body weight (PegIntron^®^, Merck Sharp & Dohme Corp.) was administered as a weekly subcutaneous injection, and a weight-based RBV dose of 15 mg/kg (Copegus^®^, Roche Pharmaceuticals, or Rebetol^®^, Merck Sharp & Dohme Corp.) was given orally twice daily for non-cirrhosis patients while RBV dose 800 mg was given orally twice daily for cirrhosis patients.

Patients infected with HCV-GT1, GT2, GT4, and GT6 without cirrhosis were treated with a fixed-dose combination of SOF 400 mg + LDV 90 mg in a single-tablet (Ledvir^®^, Mylan Laboratories Limited) orally once daily in the morning for 12 weeks while those with cirrhosis were treated with a fixed-dose combination of SOF 400 mg + LDV 90 mg orally once daily in the morning plus a weight-based RBV dose of 15 mg/kg given orally twice daily for 12 weeks. However, the attending physician had discretion to adjust the RBV dose as appropriate for each patient during the follow-up period.

### Data collection and outcomes assessment

The start date of the given DAAs was considered as the baseline of treatment in this study. Demographics and baseline characteristics (e.g., age, gender, body mass index (BMI), degree of liver fibrosis), laboratory data (e.g., liver biochemical tests, serum creatinine, complete blood count, and coagulation tests) of patients were retrospectively collected from medical records. For patients receiving Peg-IFN, thyroid function test was checked at pre-treatment visit. All the patients had follow-up once every 4 weeks and were assessed for adverse events (AEs) and compliance with the treatment.

HCV-genotype was determined by direct sequencing of the HCV core gene using the ABI3500 Genetic Analyzer instrument (Applied Biosystem, CA, USA). Serum HCV RNA was measured using real-time HCV assays by polymerase chain reaction using the COBAS^®^ AmpliPrep/COBAS^®^ TaqMan^®^ HCV Test (Roche Molecular Diagnostics, CA, USA), which quantified HCV RNA with a limit of detection of 15 IU/mL, and HCV RNA quantification was performed at baseline and week 12 after completion of therapy.

The primary outcome was to assess the overall SVR rate, which was defined as serum HCV RNA undetectable at 12 weeks after the end of the treatment, after SOF-based therapy. Secondary outcomes were to analyze the associated factors of SVR and evaluate the treatment-related AEs.

### Statistical analysis

Statistical analyses were performed using the SPSS software version 17.0 (SPSS Inc., Chicago, IL). Demographic data and baseline characteristics were analyzed using descriptive statistics, and categorical variables were compared using the Chi-square test or Fisher exact test as appropriate. Continuous variables were compared using the Independent t-test or the Mann-Whitney U test. All statistical examinations were two-tailed with a p-value <0.05 considered statistically significant.

## Results

### Baseline characteristics of study population

Data of 232 patients with chronic HCV infection were retrieved for analysis; however, twelve patients were excluded due to loss to follow-up and missing data, and another thirty-five due to their having HBV or HIV co-infection. A total of 185 patients who completed SOF-based treatment were therefore included in this study. Males accounted for 67.0% of participants, and the mean age was 54.61 years (SD 9.62). With regard to genotype distribution, GT1 was predominant at 40.0%, followed by GT3 at 37.8%, and GT6 at 22.2%. High baseline viral load, defined as HCV RNA ≥6,000,000 IU/mL, was noted in 25 patients (13.5%). Overall, 59.5% of patients had cirrhosis, and most of these were compensated (CTP Class A 82.7% and CTP Class B 17.3%). A total of 35 patients (18.9%) were treatment-experienced. Demographics and baseline characteristics of the enrolled patients are reported in Table 1. The details of the treatment administered to the study population, according to HCV genotype, are outlined in Table 2.

**Table 1:** Demographic and laboratory data of patients.

Parameters	Total (N = 185)
Age (years), mean ± SD	54.61 ± 9.62
Male sex, n (%)	124 (67.0)
BMI (kg/m^2^), mean ± SD	24.50 ± 4.31
HCV genotype	
1, n (%)	74 (40.0)
3, n (%)	70 (37.8)
6, n (%)	41 (22.2)
HCV RNA (IU/mL), median (range)	1,240,000 (5,824–25,800,000)
<6,000,000 IU/mL, n (%)	160 (86.5)
≥6,000,000 IU/mL, n (%)	25 (13.5)
Liver fibrosis stages	
Significant to advanced fibrosis (F2-3), n (%)	75 (40.5)
Cirrhosis (F4), n (%)	110 (59.5)
CTP Class A, n (%)	91 (49.2)
CTP Class B, n (%)	19 (10.3)
MELD score (among cirrhosis), median (range)	8 (6–15)
Prior treatment history	
Treatment-naïve, n (%)	150 (81.1)
Treatment-experienced, n (%)	35 (18.9)
Biochemical markers	
Total bilirubin (mg/dL), median (range)	0.74 (0.11–2.54)
AST (U/L), median (range)	73 (13–336)
ALT (U/L), median (range)	76 (11–366)
ALP (U/L), median (range)	88 (38–319)
Albumin (g/dL), median (range)	4.30 (2.30–5.50)
INR, median (range)	1.07 (0.87–2.03)
WBC (/mm^3^), median (range)	6,190 (2,570–12,170)
Hemoglobin (g/dL), mean ± SD	13.54 ± 1.70
Platelet (x10^9^/L), mean ± SD	172.46 ± 75.04
Creatinine (mg/dL), mean ± SD	0.91 ± 0.23

BMI: Body mass index; HCV: Hepatitis C virus

CTP: Child-Turcotte-Pugh; MELD: Model for end-stage liver disease

AST: Aspartate aminotransferase; ALT: Alanine aminotransferase; ALP: Alkaline phosphatase

INR: International normalized ratio.

**Table 2:** Treatment regimen administered to the patients.

Treatment regimen	Genotype-1 (n = 74)	Genotype-3 (n = 70)	Genotype-6 (n = 41)
SOF+Peg-IFN+RBV 12 weeks	0 (0)	70 (100.0)	0 (0)
SOF+LDV 12 weeks	34 (45.9)	0 (0)	18 (43.9)
SOF+LDV+RBV 12 weeks	40 (54.1)	0 (0)	23 (56.1)

SOF: Sofosbuvir; Peg-IFN: Pegylated interferon; RBV: Ribavirin; LDV: ledipasvir.

### Efficacy assessment and associated factors of SVR

**Fig 1:**
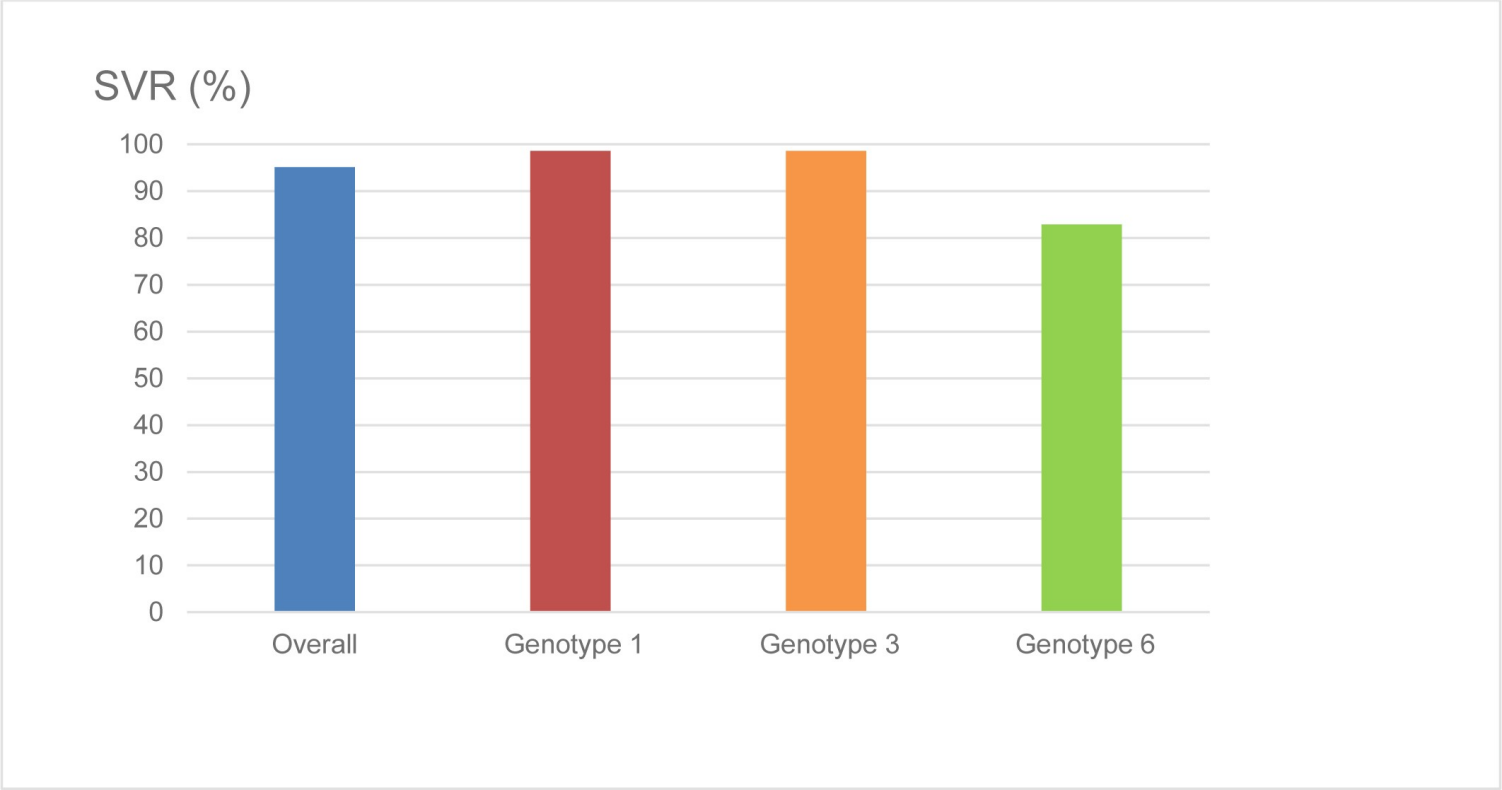
The sustained virological response among patients receiving SOF-based regimens according to HCV genotypes.

Overall, SVR was achieved by 176 of the 185 patients (95.1%) who were treated with SOF-based regimens (Fig 1), and HCV genotype was found to be the only factor associated with SVR (p = 0.001). GT6 patients had lower SVR rates compared to patients infected with GT1 and GT3 (82.9%, 98.6% and 98.6% respectively) (Fig 1) and no association was found between SVR and the other factors (p>0.05) such as gender, age ≤65 and >65 years, BMI <25 and ≥25 kg/m^2^, presence or absence of cirrhosis, baseline HCV viral load, or prior treatment history. (Table 3) Further subgroup analysis in genotype 6 patients did not show any baseline factors significantly correlated with treatment failure. (Table 4)

**Table 3:** Factors associated with sustained virological response.

Factors		SVR (n = 176)	Non-SVR (n = 9)	p-value
Gender	Male	119 (96.0)	5 (4.0)	0.480
	Female	57(93.4)	4 (6.6)	
Age	≤65	156 (95.1)	8 (4.9)	1.000
	>65	20 (95.2)	1 (4.8)	
BMI	<25	70 (97.2)	2 (2.8)	0.486
	≥25	106 (93.8)	7 (6.2)	
Liver fibrosis stage	Non-cirrhosis	74 (98.7)	1 (1.3)	0.086
	Cirrhosis	102 (92.7)	8 (7.3)	
Genotype	1	73 (98.6)	1 (1.4)	0.001*
	3	69 (98.6)	1 (1.4)	
	6	34 (82.9)	7 (17.1)	
Viral load	Low	152 (95.0)	8 (5.0)	1.000
	High	24 (96.0)	1 (4.0)	
Prior treatment history	Naïve	145 (96.7)	5 (3.3)	0.067
	Experienced	31 (88.6)	4 (11.4)	

SVR: Sustained virological response; BMI: Body mass index.

**Table 4:** Factors associated with sustained virological response in genotype 6 patients.

Factors		SVR (n = 34)	Non-SVR (n = 7)	p-value
Gender	Male	24 (85.7)	4 (14.3)	0.659
	Female	10 (76.9)	3 (23.1)	
Age	≤65	25 (80.6)	6 (19.4)	0.660
	>65	9 (90.0)	1 (10.0)	
BMI	<25	13 (86.7)	2 (13.3)	1.000
	≥25	21 (80.8)	5 (19.2)	
Liver fibrosis stage	Non-cirrhosis	16 (94.1)	1 (5.9)	0.207
	Cirrhosis	18 (75.0)	6 (25.0)	
Viral load	Low	27 (81.8)	6 (18.2)	1.000
	High	7 (87.5)	1 (12.5)	
Prior treatment history	Naïve	27 (90.0)	3 (10.0)	0.069
	Experienced	7 (63.6)	4 (36.4)	

SVR: Sustained virological response; BMI: Body mass index.

### Treatment tolerability and adverse events

With regard to tolerability, no patient had treatment discontinuation and almost all (96%) had more than 80% adherence to prescribed medication. No serious adverse events were reported in the present study, and details of AEs are shown in Table 5; fatigue was the most common during treatment, followed by anemia and headache. Of the 14 patients (7.6%) who developed anemia, ribavirin dose reduction and blood transfusion were required in six and one patients respectively.

**Table 5:** Adverse events of therapy.

	SOF+Peg-IFN+RBV (n = 70)	SOF+LDV (n = 52)	SOF+LDV+RBV (n = 63)	Total (N = 185)
Fatigue	17 (24.3)	4 (7.7)	6 (9.5)	27 (14.6)
Anemia	9 (12.9)	0 (0.0)	5 (7.9)	14 (7.6)
Headache	6 (8.6)	3 (5.8)	4 (6.3)	13 (7.0)
Flu-like symptoms	12 (17.1)	0 (0.0)	0 (0.0)	12 (6.5)
Nausea	7 (10.0)	1 (1.9)	2 (3.2)	10 (5.4)
Neutropenia	6 (8.6)	0 (0.0)	0 (0.0)	6 (3.2)

SOF: Sofosbuvir; Peg-IFN: Pegylated interferon; RBV: Ribavirin; LDV: ledipasvir.

## Discussion

In this study, the author analyzed the real-world efficacy of SOF-based regimens in Thai chronic hepatitis C patients. Our study showed an overall SVR rate of 95.1%, which is not very different from previous clinical trials and systematic reviews.[14–17, 26–29] Also, the current study found that apart from GT6, SVR rates were not significantly different among different baseline factors, included patients’ age, gender, BMI, liver fibrosis stage, viral loads and prior treatment history.

Hepatitis C virus GT6 is predominantly encountered in Asia, including Thailand.[5,30] Data for treatment efficacy of SOF-based regimens on GT6, however, are limited and based on studies with small numbers of patients. A study from New Zealand, an open-label clinical trial, included twenty-five GT6-infected patients, who were treated with 12 weeks of SOF+LDV and achieved 96% SVR.[31] Another, the U.S. community-based real-world cohort study, evaluated the effectiveness of SOF+LDV in 65 patients (98.5% were Vietnamese) with HCV GT6. Most of these patients were treated with a 12-week regimen, and an overall SVR rate of 95% was achieved.[32]

In contrast, an open-label cohort study from Myanmar revealed a poor SVR rate of only 64% for patients with GT6 who were treated with SOF+LDV for 12 weeks, and an even lower rate of 42% in those with cirrhosis.[33] Hlaing et al. have postulated that the lower SVR rate in their study compared to the study from New Zealand was a result of different prevalence levels of cirrhosis and variations in the subtypes of GT6 between the study populations. In an in vitro study, the NS5A inhibitor LDV proved active against HCV GT6a with 50% effective concentration (EC_50_) values of 1.1 nmol/L, while it has relatively less antiviral activity against GT6e with EC_50_ values of 264 nmol/L.[34] Among patients with GT6, a total of 89% of patients in the study from Myanmar had GT6c-l (6c to 6l) which comprises GT6e subtype. GT6a was not identified in this study, while 68% of patients had GT6c-l and 32% had GT6a or 6b in the study from New Zealand. Similarly, an unsatisfactory response in GT6 patients (SVR rate of 89.2%) to the SOF+LDV (± RBV) demonstrated in our study might be explained by the heterogeneity of GT6 subtypes, resulting in diverse outcomes across different studies.[31–33] Currently, the AASLD-IDSA and APASL recommend SOF+LDV (± RBV) for treatment of GT6 patients; however, our data suggest that this regimen may be inadequate. In the era of widely accessible DAAs, possibly newer, more effective regimens such as SOF plus velpatasvir could be a preferable treatment option.[35]

The triple-drug therapy (SOF+Peg-IFN+RBV), despite higher incidences of AEs, was a very potent regimen for GT3 patients with a total of 98.6% patients in our cohort achieving SVR, and this is similar to the rate previously reported (SVR rate of 98.5%) in the study from Myanmar. In a previous real-world cohort study of 24 weeks of Peg-IFN+RBV combination therapy in GT3 patients, an SVR rate of 69.7% was achieved. In that study, the author found that one of the factors associated with treatment failure was non-compliance with more than 80% of all prescribed medication, and this accounted for 25.2% in that study.[36] Unlike the findings of our present study, the triple-drug (SOF+Peg-IFN+RBV) regimen for 12 weeks was generally well tolerated, although 55.7% of patients had cirrhosis. None had treatment discontinuation and almost all had good adherence to therapy. Our findings suggest that add-on SOF in the shortened 12-week course of Peg-IFN+RBV yields fewer discontinuations and facilitates treatment completion compared to historic regimens of 24-week duration. To date, the APASL still recommends the combination of Peg-IFN+RBV±DAAs in countries with limited resources. Our data showed impressive responses to this therapy, and we favor maintaining interferon-containing regimens as the standard of care in GT3 patients in our country until a more cost-effective treatment is available.

During the interferon era, HCV GT1 was considered the most difficult genotype to treat, with combined Peg-IFN+RBV treatment for 48 weeks leading to about 40% SVR.[6] Since the DAAs against HCV GT1 have been developed, the combination of those drugs without Peg-IFN has improved SVR rates and shortened treatment duration. From our cohort, patients with GT1 treated with SOF+LDV (± RBV) for 12 weeks had SVR rates of 98.6%, comparable to the efficacy previously demonstrated in many clinical trials.[15,28,29] Currently, the AASLD-IDSA and APASL recommend SOF+LDV (± RBV) for 12 weeks to treat GT1, therefore our current experience confirms the validity of those recommendations in a real-world setting.

With regard to baseline characteristics that predicted response to treatment, bivariate analysis showed that GT6 was significantly correlated with a lower response rate to SOF-based therapy compared with GT1 and GT3 patients (p = 0.001). (Table 3) However, we did not find other factors associated with treatment outcome to incorporate into the multivariable logistic regression analysis, and our data may not be powerful enough to confirm this conclusion; nevertheless, the study from Myanmar also found that GT6 was a negative independent predictor of response to SOF-based regimen, identified by multivariable logistic regression analysis with an OR of 0.35 (95%CI: 0.16–0.79, p = 0.012),[33] so that, in clinical practice, when choosing the best treatment for an individual patient, baseline patient characteristics, especially genotype, should be taken into account.

The common AEs found in all treatment groups were fatigue (14.6%) and headache (7.0%). Among patients taking SOF+Peg-IFN+RBV, flu-like symptoms and neutropenia, well-known side-effects of Peg-IFN; were found in a minority of patients, furthermore, they were mild and did not lead to discontinuation of treatment in any of the study population. In addition, RBV-associated anemia was successfully managed through RBV dose reductions and only one patient who took SOF+Peg-IFN+RBV required a blood transfusion. These data underscore the fact that the shortened 12-week duration of SOF-based therapy, even in patients with cirrhosis, facilitates treatment completion despite the expected side-effects of both Peg-IFN and RBV.

While our study represents the first real-world experience and provides helpful information about the efficacy and safety of SOF-based DAA regimens in the treatment of chronic HCV infection in Thai patients, our findings had several limitations. Firstly, the observational nature of the study design was a limiting factor; furthermore, all patients were Thai and all were treated at a single tertiary center in Bangkok, so these findings may not be generalizable to other patient populations. In addition, while our sample size was large enough to evaluate the SOF-based treatment efficacy in a real-world setting, we acknowledge that our study was not designed to estimate variations in outcomes between different treatment strategies, such as triple-drug therapy (SOF+Peg-IFN+RBV) or SOF+LDV regimen with RBV or without RBV, and no conclusion can therefore be drawn with respect to this important aspect. There is another concern about the over-diagnosis of cirrhosis (59.5%) in our study population, that based on VCTE ≥13.5 kPa according to the Thailand practice guideline for the management of chronic hepatitis C 2018 and this proposed cut-off (11.9–14.8 kPa) offer a PPV ranging from 52% to 85%. Therefore, VCTE above this cut-off may not be enough to confirm the presence of cirrhosis, reflects from the baseline laboratory results of our patients, the median serum albumin and platelet count were 4.3 g/dL and 172 x 10^9^/L, respectively, which are corresponding to mild-to-moderate fibrosis. Finally, we hypothesized that the poorer response to SOF-based therapy in GT6 patients might be due to the heterogeneity of GT6 subtypes, and that our GT6 population may have had the important components of GT6e subtype similar to the study from Myanmar^(33)^; however, we did not have the details about HCV subtypes in our current data to support this hypothesis.

In conclusion, our study demonstrated that Sofosbuvir-based regimens in the treatment of patients with chronic HCV infection were highly efficacious with excellent safety and tolerability profiles in a real-world setting. Whether or not such regimens are adequate for treatment of all genotype 6 patients needs to be further explored.
